# Intensity modulated radiation therapy with stereotactic body radiation therapy boost for unfavorable prostate cancer: five-year outcomes

**DOI:** 10.3389/fonc.2023.1240939

**Published:** 2023-11-23

**Authors:** Michael Carrasquilla, Tamir Sholklapper, Abigail N. Pepin, Nicole Hodgins, Siyuan Lei, Abdul Rashid, Malika Danner, Alan Zwart, Grecia Bolanos, Marilyn Ayoob, Thomas Yung, Nima Aghdam, Brian Collins, Simeng Suy, Deepak Kumar, Ryan Hankins, Keith Kowalczyk, Nancy Dawson, Sean Collins

**Affiliations:** ^1^ Department of Radiation Oncology, Georgetown University Hospital, Washington, DC, United States; ^2^ Department of Urology, Einstein Healthcare Network, Philadelphia, PA, United States; ^3^ Department of Radiation Oncology, University of Pennsylvania, Philadelphia, PA, United States; ^4^ School of Medicine, Georgetown University Hospital, Washington, DC, United States; ^5^ Department of Radiation Oncology, Beth Israel Deaconess Medical Center, Boston, MA, United States; ^6^ Department of Radiation Oncology, Tampa General Hospital, University of South Florida, Tampa, FL, United States; ^7^ Biomedical Research Institute, North Carolina Central State, Durham, NC, United States; ^8^ Department of Urology, Georgetown University Hospital, Washington, DC, United States; ^9^ Department of Medical Oncology, Georgetown University Hospital, Washington, DC, United States

**Keywords:** prostate cancer, SBRT, IMRT, CyberKnife, SBRT boost

## Abstract

**Purpose:**

Intensity-modulated radiation therapy (IMRT) with brachytherapy boost for unfavorable prostate cancer has been shown to improve biochemical relapse-free survival compared to IMRT alone. Stereotactic body radiation therapy (SBRT) is a less-invasive alternative to brachytherapy. Early outcomes utilizing SBRT boost suggest low rates of high-grade toxicity with a maintained patient-reported quality of life. Here, we report the 5-year progression-free survival (PFS) and prostate cancer-specific survival (PCSS) of patients treated with IMRT plus SBRT boost.

**Materials and methods:**

Between 2008 and 2020, 255 patients with unfavorable prostate cancer were treated with robotic SBRT (19.5 Gy in three fractions) followed by fiducial-guided IMRT (45–50.4 Gy) according to an institutional protocol. For the first year, the patient’s PSA level was monitored every 3 months, biannually for 2 years, and annually thereafter. Failure was defined as nadir + 2 ng/mL or a rising PSA with imaging suggestive of recurrence. Detection of recurrence also included digital rectal examination and imaging studies, such as MRI, CT, PET/CT, and/or bone scans. PFS and PCSS were calculated using the Kaplan–Meier method.

**Results:**

The median follow-up period was 71 months. According to the NCCN risk classification, 5% (13/255) of the patients had favorable intermediate-risk disease, 23% (57/255) had unfavorable intermediate-risk disease, 40% (102/255) had high-risk disease, and 32% (83/255) had very high-risk disease. Androgen deprivation therapy was administered to 80% (204/255) of the patients. Elective pelvic lymph node IMRT was performed in 28 (10%) patients. The PFS for all patients at 5 years was 81% (favorable intermediate risk, 91%; unfavorable intermediate risk, 89%; high-risk, 78%; and very-high risk, 72%). The PCSS for all patients at 5 years was 97% (favorable intermediate risk, 100%; unfavorable intermediate risk, 100%; high risk, 100%; and very high risk, 89%).

**Conclusion:**

The incidence of failure following IMRT plus SBRT for unfavorable prostate cancer remains low at 5 years.

## 1 Introduction

In total, 248,500 men were diagnosed with prostate cancer in 2022, making it the most prevalent malignancy among men in the United States (1). Approximately 20% of patients newly diagnosed with prostate cancer present with high-risk disease, with an expected increase in the proportion due to decreased PSA screening (2). Radiotherapy is the first-line treatment for patients with prostate cancer. Several randomized prospective trials have demonstrated that dose-escalated radiotherapy results in improved biochemical free survival in patients with intermediate- and high-risk diseases (3–5). The development and improvement of image-guided radiation therapy (IGRT) (6) and low-dose-rate brachytherapy boost (7, 8) have further improved outcomes in these patients. SBRT offers the potential for better results.

Large radiation fraction sizes have been shown to likely confer a radiobiologic advantage in the treatment of prostate adenocarcinoma (9), supporting the use of high-dose rate (HDR) brachytherapy as a boost to external beam radiation therapy (EBRT) for intermediate- and high-risk patients. Several retrospective reviews have reported 5-year biochemical control rates of 89%–93% (10–12) and 69%–83% (10–13) for intermediate- and high-risk prostate cancer, respectively. These results have also been shown prospectively (14–16). Not unexpectedly, these improved outcomes are achieved with an increased risk of significant long-term genitourinary toxicities including urethral stricture and incontinence (16–18).

SBRT efficiently delivers high doses of radiation without invasive procedures. We have examined the use of stereotactic body radiation therapy (SBRT) as a boost to image-guided intensity-modulated radiation therapy (IMRT) for the treatment of patients with unfavorable risk prostate cancer to maximize the benefits of administering high doses per fraction, and to minimize the short- and long-term consequences. Previously, we have reported early outcomes of this treatment modality, including: 3-year biochemical recurrence free survival, acute toxicity, 3-year toxicity and quality of life (18–20). Our reports suggest that this treatment approach has a minimal impact on long-term quality of life and provides excellent early disease outcomes (19, 20). These results have been demonstrated in several other studies (21–24). Here, we report the five-year progression-free survival (PFS) and prostate cancer-specific survival (PCSS) in a cohort of patients treated at Georgetown University Medical Center with this treatment approach.

## 2 Methods

### 2.1 Patient selection

Patients with histologically confirmed adenocarcinoma of the prostate and intermediate, high, or very high-risk prostate cancer according to the National Comprehensive Cancer Network (NCCN) risk grouping were included in the study. Several patients (~5%) were categorized as having favorable intermediate risk according to the NCCN criteria, as they were treated according to this institutional protocol prior to the establishment of the criteria. All patients underwent a bone scan and pelvic imaging (pelvic CT and/or MRI) as clinically indicated according to the national guidelines. The exclusion criteria were clinically involved lymph nodes, bone metastases or prior pelvic radiotherapy. Androgen deprivation therapy was considered for all unfavorable intermediate, high, and very high-risk patients and was ultimately administered at the discretion of the treating physicians. Institutional IRB approval was obtained for this study (IRB 09-510).

### 2.2 SBRT treatment planning and delivery

**Figure 1:**
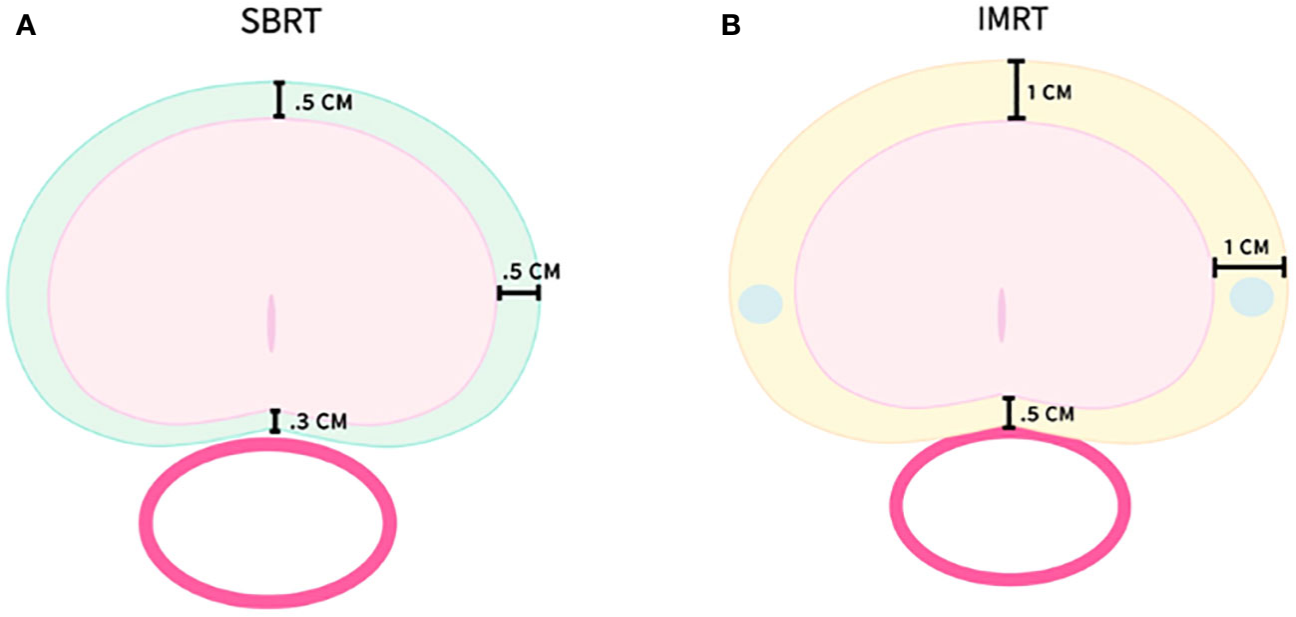
SBRT and IMRT volumes with PTV expansions. **(A)** PTV1–SBRT treatment volume. **(B)** PTV2–IMRT treatment volume.

Treatment planning and delivery have been previously reported (20). Briefly, all patients received fiducials placed in the prostate prior to treatment planning. The patients then underwent MRI and thin-cut (1.25 mm) CT scan of the pelvis with an empty bladder. If a patient had a contraindication to MRI, a CT urethrogram at the time of CT simulation was employed as an alternative imaging approach to identify the location of the prostatic apex (25). Patients were advised to adhere to a low-gas, low-motility diet starting at least five days prior to all treatment planning imaging and treatment delivery. An enema was administered 1 h–2 h prior to imaging and SBRT. The CT and MR images were fused for treatment planning. The clinical target volume (CTV1) included the prostate, areas of radiographic extracapsular extension and seminal vesicles proximal to the point of separation. The SBRT planning target volume (PTV1) was equal to the CTV1 expanded 3 mm posteriorly and 5 mm in all other dimensions (**Figure 1A**). The prescription dose was 19.5 Gy to PTV1 delivered in three fractions of 6.5 Gy over 3–5 days. The prescription isodose line was limited to ≥75%, which limited the maximum prostatic urethral dose to 133% of the prescription dose. The rectum, bladder, penile bulb, and membranous urethra were contoured and evaluated using dose-volume histogram analysis during treatment planning using Multiplan (Accuray Inc., Sunnyvale, CA, USA) inverse treatment planning.

Following SBRT, IMRT treatment was initiated the following week. Most patients (89%) were treated to the prostate alone with a more generous PTV2 including a margin of 1.0 cm around CTV1, except at the rectal interface where a margin of 0.5 cm was added (**Figure 1B**). A minority of patients (11%) were treated in the prostate and pelvic lymph node basins with PTV3 encompassing the previously noted expansion (PTV2) with the addition of the RTOG consensus lymph node basins (26). Daily doses of 1.8 Gy were delivered to PTV2/PTV3 5 days a week to a total dose of 45 Gy–50.4 Gy in 25–28 fractions. One hundred percent of PTV2/PTV3 received at least 95% of the prescription dose, and 5% of the volume received no more than 105% of the prescription dose. Dose and volume constraints as well as the process of combining the IMRT and SBRT plans into a radiobiologically equivalent dose-volume histogram (DVH) have been previously described (19, 20, 27).

### 2.3 Linear-quadratic transformation of a sample combined physical IMRT plus SBRT boost dose-volume histogram to a radiobiologically equivalent DVH

A radiobiologically equivalent dose of DVH was generated by adding doses in 2 Gy equivalents for IMRT and SBRT plans from a sample patient (28). Cumulative DVHs were extracted from the treatment planning software and converted to radiobiologically equivalent DVHs using MIM software (MIMvista Corporation). An α/β ratio of 1.5 was utilized to transform the target volume doses (GTV and PTV), and an α/β ratio of 3 was used to transform doses for all other organs at risk (OAR).

### 2.4 Follow-up

Patients were assessed at the start of and one month after therapy, every 3 months for the first year, and every 6 months thereafter. The patient’s PSA level was monitored every 3 months during the first year, biannually for 2 years, and annually thereafter. Failure was defined using the nadir + 2 ng/mL definition or a rising PSA level after long-term nadir with imaging suggestive of disease recurrence. Detection of recurrence included digital rectal examination, imaging studies such as MRI, CT, PET/CT (sodium-F, PSMA, and Axumin), and/or bone scan. PFS and PCSS were calculated using the Kaplan–Meier method.

## 3 Results

Between 2008 and 2020, 255 patients with intermediate- and high-risk prostate cancer were treated using an institutional IMRT plus SBRT boost protocol. The median follow-up was 5.9 years (range, 2–12 years). The patient characteristics are shown in **Table 1**. The median patient age was 70 years (IQ range, 65–75 years). The median pretreatment prostate-specific antigen (PSA) was 10.7 ng/ml. (IQ range, 6.3 ng/ml–20.2 ng/ml). A total of 29 (11%) patients had a PSA level >40 ng/ml prior to treatment. According to the NCCN Risk Classification, 5% were diagnosed with favorable intermediate-, 23% with unfavorable intermediate-, 40% with high-risk disease and 32% with very high-risk disease, respectively. Approximately 10% of the patients received prophylactic radiation to the RTOG consensus pelvic lymph node basins. Approximately 80% of patients received androgen deprivation therapy (ADT).

**Table 1:** Patient characteristics and treatment specifics.

		Count	(%)
Age, years			
	Median, IQR	70.3	(65.7, 75.2)
PSA, ng/mL			
	Median, IQR	10.7	(6.3, 20.2)
	<10	114	44.7%
	“10–20”	71	27.8%
	>20	63	24.7%
T-Stage			
	T1	100	39.2%
	T2	134	52.5%
	T3a	3	1.2%
	T3b	4	1.6%
	T4	1	0.4%
Gleason Grade Group			
	1	15	5.9%
	2	50	19.6%
	3	43	16.9%
	4	81	31.8%
	5	66	25.9%
NCCN Risk Group			
	Favorable Intermediate	13	5.1%
	Unfavorable Intermediate	57	22.4%
	High Risk	102	40.0%
	Very High Risk	83	32.5%
Pelvic Lymph Node Treatment			
	Yes	28	11.0%
	No	227	89.0%

**Figure 2:**
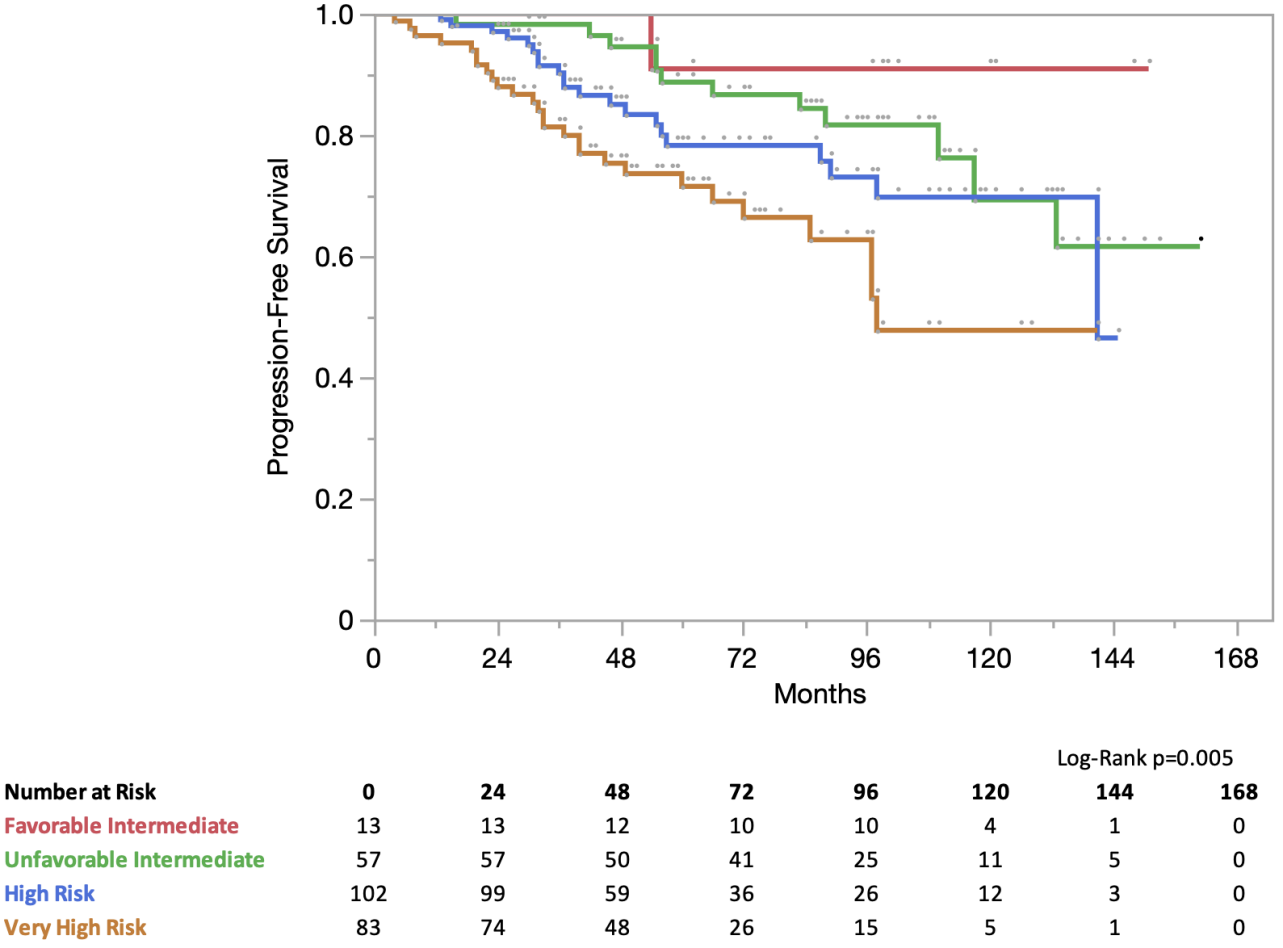
Progression free survival (PFS) stratified by NCCN Risk Group.

**Figure 3:**
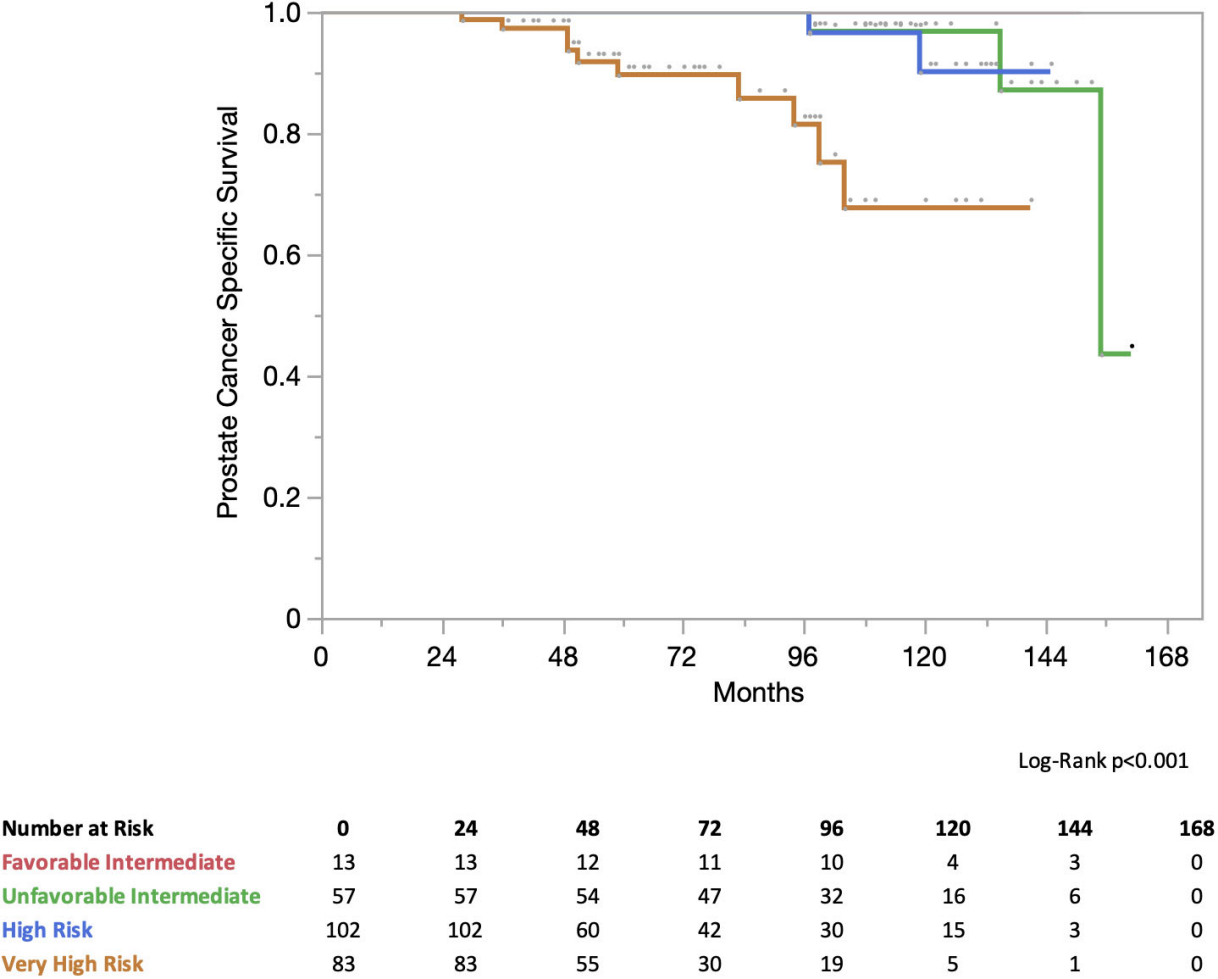
Prostate cancer specific survival (PCSS) stratified by NCCN risk groups.

At 5 years, progression-free survival (PFS) for all patients was 81%. In the NCCN risk group the PFS was 5 years for favorable intermediate risk: 91% for unfavorable intermediate risk, 89% for high-risk, 78% for high-risk, and 72% for very high-risk (**Figure 2**). At 5 years, the prostate cancer-specific survival (PCSS) for all patients was 97%. In the NCCN risk group, the PCSS at 5 years for favorable intermediate risk was 100%, unfavorable intermediate risk was 100%, high-risk was 100%, and very high-risk was 89% (**Figure 3**).

## 4 Discussion

This study aimed to assess progression-free survival and prostate cancer-specific survival outcomes in patients with prostate cancer patients receiving IMRT plus SBRT boost. ASCENDE-RT examined men with intermediate- and high-risk prostate cancer randomized to receive pelvic irradiation followed by dose-escalated EBRT boost or LDR brachytherapy boost. In this trial, bPFS was significantly improved in the LDR brachytherapy arm compared to the EBRT arm (83% vs. 62% 9-year bPFS). Patients in the EBRT arm were twice as likely to experience biochemical failure. No significant difference was detected in the overall survival difference between the treatment arms. Importantly, the ASCENDE-RT trial reported a cumulative late grade ≥3 toxicity of 18.4% at 5-years compared with 5.2% in the EBRT arm. Specifically, LDR increases the risk of needing temporary catheterization and/or incontinence pads because of urethral strictures, urinary retention, or incontinence. SBRT boost was chosen for this study due to the potential radiobiological benefits of hypofractionation in addition to being a less invasive and toxic alternative to brachytherapy boost (18–21).

In our study, the incidence of failure following IMRT plus SBRT boost was low at 5-years. PFS and PCSS were 81% and 97% at 5-years in all patients. Unsurprisingly, PFS decreased in the high-risk groups: 91%, 89%, 78%, and 72% for favorable intermediate-risk, unfavorable intermediate-risk, high-risk, and very high-risk groups, respectively. The PCSS was 100% in favorable intermediate risk, unfavorable intermediate risk, and high-risk disease, but decreased to 89% in our very high-risk cohort. These results appear similar to those reported for brachytherapy boost, despite our very high-risk cohort, robust surveillance, and lack of prophylactic pelvic nodal irradiation.

The Phoenix definition (nadir PSA + 2 ng/mL) after radiation therapy was used to classify biochemical failure. However, the Phoenix criteria were developed for low-dose, conventionally fractionated EBRT. Compared with EBRT, PSA nadirs are lower with brachytherapy and SBRT (29, 30). An alternative criterion for classifying biochemical failure in SBRT patients has been proposed (31). As a result, our practice includes imaging before meeting the Phoenix criteria for failure. Ultimately, this could lead to lower metastasis-free survival and bRFS.

The prophylactic treatment of pelvic lymph nodes with RT has been a source of ongoing debate. The rationale has been to eradicate nodal micrometastases, with the goal of improving regional control. In GETUG-01, there was no observed benefit in event-free or OS with pelvic irradiation, although *post-hoc* analysis favored pelvic RT in patients with a <15% Roach nodal risk. More recently, POP-RT previously randomized prophylactic whole pelvic nodal RT to prostate-only radiation in 224 high-risk prostate cancer patients, with a median Roach nodal risk of 37.8%. In that study, WPRT demonstrated a higher 5-year bFFS (95% vs. 81.2%) and PFS (89.5% vs. 77.2%) than PORT. However, there was no significant difference in the 5-year overall survival (92.5% vs. 90.8%). Our study included 10% of patients who received elective nodal IMRT, many of whom had PSA >40 ng/mL, had T3+ disease, and were classified as very-high risk of NCCN. Similarly, POP-RT also included 162 patients with T3+ disease, 60 patients with >50 PSA levels, and 116 patients with high-risk disease. However, notably, the POP-RT trial utilized PSMA PET for staging in 80% of the patients, excluding patients with occult metastases. In our population study, PMSA PET was not used for staging.

Our study has several limitations. Our study was retrospective in nature and inherently limited. Our patients did not undergo fluciclovine/prostate specific membrane antigen (PSMA) PET scans for initial staging due to a lack of availability in the United States; as a result, many of the very high-risk patients in our study had occult metastases prior to treatment. Approximately 90% of patients did not receive prophylactic pelvic lymph node irradiation. Since the adoption of VMAT at our center and with improved outcomes shown in the POP-RT study, it is our current practice to treat high-risk patients with prophylactic pelvic lymph node irradiation. Our treatment approach may be unduly burdensome because of the extended (5 weeks) course of pelvic radiation used in this study. Future studies utilizing short-course pelvic radiation and directly comparing brachytherapy boost with SBRT boost are required.

## 5 Conclusion

IMRT with SBRT boost is a promising treatment option for men with unfavorable prostate cancer. The incidence of failure following IMRT plus SBRT for unfavorable prostate cancer remains low at 5 years. Future studies directly comparing brachytherapy boost with SBRT boost are warranted, with endpoints including disease control and patient-reported quality of life.

## Data availability statement

The datasets presented in this article are not readily available because of patient confidentiality. Requests to access the datasets should be directed to MC, michael.a.carrasquilla@gunet.georgetown.edu.

## Ethics statement

The studies involving humans were approved by Georgetown University Hospital. The studies were conducted in accordance with the local legislation and institutional requirements. The participants provided their written informed consent to participate in this study.

## Author contributions

All authors contributed to the treatment of patients, conceptualization of the study, drafting and editing the manuscript and/or follow up of patients and acquisition of continued follow up data over 5 years. All authors contributed to the article and approved the submitted version.
